# Mr. Cuming, in Reply to Dr. Scott

**Published:** 1804-03-01

**Authors:** Ralph Cuming

**Affiliations:** Romsey, Hants


### *To the Editors of the Medical and Physical Journal.*

Gentlemen,

Being of opinion that your conduct, as Editors of so interesting a work as the Medical and Physical Journal, cannot be influenced either by favour or partiality; I beg you will act as liberally towards me, as you have done to Dr. Scott, in allowing me to reply to his Strictures on my Observations relative to the Efficacy of Zincum Vitriolatum in the Cure of Agues.

The word specific seems to have made a serious impression on the Doctor; he remarks, specifics are so fashionable, that the profession of a physician is nearly at an end. —A little farther on, he says, the word specific I very much dislike, to me the sound is *empirical*; and I can assure him, to me his observation appears extremely harsh and unbecoming; if any part of my paper savored of quackery, his observation must convey, tacitly, censure on the Editors as well as the author; and if the former had been been sensible of this, the reproof would have been just. But it remains with me to exculpate both them and myself, from an imputation as gross as it is indecent; and the Doctor should recollect that few nostrum-mongers are fond of exposing their practice in print. The Doctor may take my word for it, if he chooses, that the remarks made in my paper of No. 58, are the result of unerring observation and experience.

I have to request my reader will do me the favor to refer to that part of my paper, which proved so very objectionable to the mind's eye of the Doctor, and he will there find, if he judges liberally, from the manner in which I made use of the word specific, that it does not convey the idea of its being an infallible remedy; no man, whose judgment is not grossly perverted, could for a moment suppose so. I do not assert that the Doctor laboured under such an infirmity; no, I would judge more charitably, and suppose it arose from inattention to the subject. It will be easily perceived that I do not wish to give bark more credit than experience teaches me it deserves. What person conversant with medicine has not heard it remarked, that bark was a specific in agues? And medical authors tell you, that such a quantity will be requisite to cure an intermittent of such a type, whether quotidian, tertian, or quartan; but when exceptions are made to data well established, for the purpose of getting certain things undeservingly into disrepute, such conduct must in some degree be considered fastidious. I speak here only as far as relates to the word specific, for if lexicographers were of the opinion the Doctor is, the word would be expunged from the English language; when this happens the author need not be afraid of the critic, for then, in all probability, it will be forgotten.

I think with the Doctor, that few medicines can be honoured with such an epithet, but surely one may merit it more than another; and so long as the word is retained in our language, there can be little harm in observing that one medicine is entitled to it more than another, when it cures what the other could not; and this is the only meaning I intended the word to convey, which I trust every impartial person will readily perceive, and think with me that the word empirical borders on severity.

The Doctor acknowledges, though somewhat reluctantly, that he has a good opinion of white vitriol in the cure of agues, for he observes it answered the intention *pretty well.* Surely

Surely if it cured when other remedies failed, a better word than *pretty* might have been used; but he says, "I thought cuprum ammoniacum a better medicine," which I believe has been expunged from some Pharmacopœias, being considered by men eminent in their profession, a remedy fraught with the greatest danger; and I cannot find it in the Edinburgh Dispensatory, a book which he has quoted respecting white vitriol. He appears to recommend zincum calcinatum as preferable, and gives a formula for its exhibition in the margin, of *one grain* twice a day. He surely only means this as an auxilliary, from the smallness of the quantity, to be taken in twenty four hours; but I must here observe, under the head of zinci vitriolati purificatio, in the very book which he has quoted, that this medicine is considered preferable to the calx zinci, in cases where its powers are indicated. The compilers of this work do not appear to have known that zincum vitriolatum possessed the virtue of curing agues per se; and if there is any merit in promulgating to the world a remedy which stands high in my opinion, it cannot be the intention of the Doctor to detract from it, by laying in a claim for orginality; a claim which I never presumed to offer: but let Virgil speak for me,

"Haud equidem tali me dignor honore."

And I believe those gentlemen who are willing by experiment to decide whether the calx zinci or zincum vitriolatum deserves the preference, will find the result of their enquiries to be greatly in favour of the latter, though I must acknowledge I never gave the former a trial, or ever deemed it worthy being put in competition with the medicine I still so strenuously recommend.

The Doctor says, his patients could never take the quantity of white vitriol mentioned by me; this is very possible, I have known a small quantity effect a cure; but when trifling doses did not answer the intention, I always pushed it to the length mentioned in my paper, without producing vomition or any bad consequence; if the stomach was ever affected, a little mint water, or any other cordial, always relieved the patient. Every medical gentleman will of course be guided by the feelings of the patient, and his own discretion will tell him when to desist; the road between timidity and rashness is not at all devious, and the former is as much to be deprecated as the latter; humanity shudders at the thought of keeping a patient labouring under the most trivial complaint, longer on the sick list than than is necessary, which I believe is often the case when medicines are administered with too sparing a hand.

In replying to Dr. Scott, I kept two objects in view; the first was to refute the opinion which he has attached to the word *specific*, for I must remark the task of writing will be difficult when circumstances render it necesary to give perception as well as matter. The last was for the purpose of exciting the attention of my medical brethren, to a remedy, which, as far as my observation goes, inclines me to suppose, is as much deserving of the epithet specific, in ague, as any other that I am acquainted with.

I am, &c.

*Romsey, Hants.*

*February* 10, 1804.

RALPH CUMING.

---

